# Success of ceftazidime–avibactam and aztreonam in combination for a refractory biliary infection with recurrent bacteraemia due to *bla*IMP-4 carbapenemase-producing *Enterobacter hormaechei* subsp. *oharae*

**DOI:** 10.1099/acmi.0.000248

**Published:** 2021-08-06

**Authors:** Genevieve McKew, John Merlino, Alicia Beukers, Sebastian van Hal, Thomas Gottlieb

**Affiliations:** ^1^​ Department of Microbiology and Infectious Diseases, NSW Pathology, Concord Repatriation General Hospital, Concord, NSW, Australia; ^2^​ Faculty of Medicine and Health, The University of Sydney, Sydney, NSW, Australia; ^3^​ Department of Microbiology and Infectious Diseases, NSW Pathology, Royal Prince Alfred Hospital, Camperdown, NSW, Australia

**Keywords:** aztreonam, *bla*IMP-4, carbapenemase, ceftazidime–avibactam, *Enterobacter hormaechei *subsp. *oharae*

## Abstract

**Background:**

Infections due to metallo-beta-lactamase (MBL)-producing organisms are becoming a significant problem, and antibiotic treatment options are limited. Aztreonam inhibits MBLs, and its use in combination with ceftazidime–avibactam (CAZ–AVI–AZT) to inhibit other beta-lactamases shows promise.

**Methods:**

A 45-year-old woman suffered from recurrent and sustained MBL (*bla*IMP-4)+ *Enterobacter cloacae* complex bacteraemia from an undrainable biliary source, and had failed nine alternative antibiotic regimens over a 5-month period. The 10th episode was successfully treated with CAZ–AVI–AZT, and she has had no further relapses. Three of the isolates underwent whole-genome sequencing (WGS) on the MiSeq platform and were analysed with the Nullarbor pipeline.

**Results:**

A layered Etest method for synergy between CAZ–AVI and aztreonam demonstrated an MIC of 2 mg l^−1^ for the combination. Isolates were identified by WGS as *Enterobacter hormaechei* subsp. *oharae *. All three of the isolates had *bla*TEM-4 ESBL, *bla*OXA-1 and *bla*ACT-25. Two of the carbapenem-resistant isolates contained *bla*IMP-4.

**Conclusion:**

While aztreonam inhibits MBLs, MBL-positive isolates often express other beta-lactamase enzymes. Avibactam inhibits ESBLs and other beta-lactamases, and its use in this case possibly contributed to therapeutic success due to inhibition of the concomitant *bla*TEM-4 in the isolates. This case demonstrates that phenotypic antimicrobial susceptibility testing (layered Etests for synergy), backed up by WGS, can produce results that allow tailored antimicrobial therapy in difficult infections. This case adds to the evidence for using CAZ–AVI–AZT in serious MBL infections.

## Introduction

We present a case where treatment with ceftazidime–avibactam and aztreonam in combination was effective in a patient with recurrent and sustained *bla*IMP-4+ *Enterobacter cloacae* complex bacteraemia from an undrainable biliary source, where alternative antibiotic treatment had failed over a 5-month period.

## Case report

A 45-year-old woman was admitted to the intensive care unit (ICU) for 5 months in December 2017 with protracted status epilepticus due to limbic encephalitis. This was complicated by diffuse intra-hepatic biliary duct dilatation and marked derangement of liver function tests, presumed to be due to antiepileptic medication, with a liver biopsy demonstrating non-inflammatory, non-steatotic hepatocyte injury of unclear aetiology. She also developed sacral pressure ulcers, deep venous thrombosis, an upper gastrointestinal bleed and cardiomyopathy. After resolution of status epilepticus, she had ongoing cognitive impairment and intermittent seizures. She was treated initially with high-dose corticosteroids.

From 2 weeks after admission, over an 8-month period, she had 10 discrete episodes of Gram-negative bacteraemia, all culturing *E. cloacae* complex (MALDI Biotyper, Bruker) (see Table 1, isolates ECI01–10). These were attributed to cholangitis. She had an endoscopic retrograde cholangiopancreatogram with stenting of the mildly dilated common bile duct, but this did not improve biliary drainage. A magnetic resonance cholangiogram demonstrated a gallstone, gallbladder wall thickening, and moderate irregularity and dilatation of the intrahepatic ducts. Computed tomography demonstrated contrast enhancement of the major ducts consistent with cholangitis. Histopathology of the common bile duct revealed a mild acute inflammatory infiltrate of the mucosa and stroma, with no malignant cells. Positron-emitted tomography revealed diffuse, moderate-to-markedly increased metabolism outlining the biliary tree in both lobes of the liver, consistent with cholangitis, without other abnormalities. She was treated for cholestasis with cholestyramine and ursodeoxycholic acid. Liver transplantation was not considered an option.

**Table 1.** Antibiotic treatment regimens, *bla*IMP-4 status and interval between septic episodes for the first nine *E. cloacae* complex bloodstream infections

Isolate no.	Date	* E. cloacae* complex antibiotic resistance	Antibiotic	Days off treatment until recurrence
ECI01	23 December 2017	Wild-type	Gentamicin → meropenem, 14 days	**29**
ECI02*	7 February 2018	*bla*IMP-4, CIP intermediate, MDR†	Ciprofloxacin, 12 days	−**2**
ECI03*	18 February 2018	MDR, except cefepime-susceptible	Meropenem, 5 days	**3**
ECI04	2 March 2018	*bla*IMP-4, MDR	PTZ‡ and amikacin, 19 days	−**6**
ECI05*	10 March 2018	*bla*IMP-4, MDR	Aztreonam, 7 days	**4**
ECI06	24 March 2018	*bla*IMP-4, MDR	Amikacin 5 days, PTZ‡ 4 days, aztreonam 14 days	**6**
ECI07	5 April 2018	MDR	Amikacin 16 days	**9**
ECI08	1 May 2018	MDR	Amikacin 2 days → meropenem 12 days	**13**
ECI09	28 May 2018	*bla*IMP-4, MDR (MER MIC 4 mg l^−1^)	Amikacin and meropenem 14 days	**36**
ECI10	18 July 2018	*bla*IMP-4, MDR	Amikacin 7 days → aztreonam and ceftazidime–avibactam 14 days	**No recurrence**

*Sequenced isolates.

†MDR, resistant to ciprofloxacin, gentamicin, cefepime, trimethoprim–sulphamethoxazole, tigecycline, nitrofurantoin.

‡Piperacillin–tazobactam.

The first episode of bacteraemia occurred in December 2017. A wild-type *E. cloacae* complex isolate was cultured. This episode was followed by six further episodes of *bla*IMP-4+ *E. cloacae* complex bloodstream infection, over 5 months beginning in February 2018, interspersed with three episodes where ESBL-producing, but *bla*IMP-4-negative, *E. cloacae* complex was isolated. All *bla*IMP-4+ isolates remained amikacin-susceptible (Vitek2, BioMérieux); meropenem Etest MICs (Biomérieux) were >16 mg l^−1^ for all isolates except for one (ECI09), at 4 mg l^−1^.

Throughout these episodes she received multiple treatments of varied duration with combinations of antibiotics, including meropenem, amikacin, aztreonam, piperacillin–tazobactam, ciprofloxacin, gentamicin and trimethoprim–sulfamethoxazole (Table 1). Despite responding clinically on each occasion, particularly when amikacin was included in treatment, the septic episodes recurred regularly, usually within 1–2 weeks of antibiotic therapy ceasing. These presented clinically with slight worsening of cognitive status, low-grade fever and gradual increase in C-reactive protein and transaminase levels, without other overt signs or symptoms of typical sepsis.

A 10th episode of *E. cloacae* complex bacteraemia (*bla*IMP-4+) occurred on 18 July 2018. To aid treatment of this episode, *in vitro* synergy testing for the combination of ceftazidime–avibactam (CAZ–AVI) and aztreonam was performed. The patient then received 14 days of CAZ–AVI 2 g/0.5 g 8-hourly in combination with aztreonam 1 g 8-hourly. She had also received amikacin 900 mg daily for the previous 7 days. Aztreonam was initially dosed at 2 g, but after a seizure, a lower dosage was used because of the risk of provoking seizures with double β-lactam therapy. She tolerated the treatment course without complications.

She has had no further recurrences during 12 months of follow-up, which included 16 separate blood culture collections. Her rectal screening samples continue to culture *bla*IMP-4+ *E.cloacae* complex.

## Phenotypic testing and whole-genome sequencing (WGS)

Phenotypic antimicrobial susceptibility testing was performed with Vitek2. The colistin broth microdilution MIC was 0.25 mg l^−1^ (MERLIN Diagnostika GmbH). Supplementary testing was performed with Etest strips and the individual MIC results were 6 mg l^−1^ for tigecycline, 128 mg l^−1^ for aztreonam and >256 mg l^−1^ for CAZ–AVI. A layered Etest method for synergy between CAZ–AVI and aztreonam demonstrated an MIC of 2 mg l^−1^ for the combination.

**Fig. 1.**
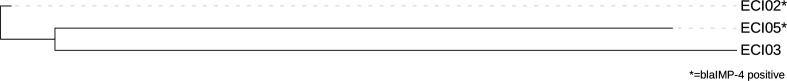
Core-genome SNP phylogeny showing the relationships between the core-genomes of isolates ECI02, ECI03 and ECI05, built using the maximum-likelihood GTR+G4 model. It demonstrates an 1120 core-genome SNP difference between ECI05 and ECI03, whilst both are more closely related to ECI02 (590 and 645 core-genome SNP differences, respectively). Notably, ECI02 and ECI05 were both *bla*IMP-4-positive, despite the differences in core-genome, and this is presumed to be due to its likely presence on the IncHI2 plasmid.

WGS was performed on the Illumina MiSeq platform and analysed with the Nullarbor pipeline [1] for three of the isolates. ECI02 was the first non-wild-type isolate, and was *bla*IMP-4 PCR-positive and multidrug-resistant (MDR). ECI03 was the first *bla*IMP-4 PCR-negative but still MDR isolate, but notably, cefepime-susceptible. ECI05 was again *bla*IMP-4 PCR-positive, and was chosen because treatment with amikacin had failed. Trimmed reads from ECI02, ECI03 and ECI05 were aligned to the reference *E. cloacae* ATCC 13047 (GenBank accession CP001918) to determine core single-nucleotide polymorphisms (SNPs) between the patient’s isolates using Snippy-core. These were aligned to infer core SNP phylogeny (maximum-likelihood GTR+G4 model) with IQTree (see Fig. 1). ECI05 and ECI03 are more closely related to ECI02 (590 and 645 core-genome SNP differences, respectively) than they are to each other (1120 core-genome SNP difference). Of note, ECI02 and ECI05 are the *bla*IMP-4-positive isolates, despite their core-genome differences. Species identification (Kraken) [2] for all three isolates was consistent with *Enterobacter hormaechei* subsp. *oharae* (part of the *E. cloacae* complex). They were found to belong to multilocus sequence type 114 (mlst 2.6, http://pubmlst.org/). Sequence data are available in the European Nucleotide Archive (accession PRJEB39176).

Plasmid replicons were detected by uploading assembled contigs (from SPAdes v3.12.0) [3] to PlasmidFinder [4]. Contigs were uploaded to the CARD database to detect antimicrobial resistance genes [5]. The isolates were MDR, and multiple antibiotic resistance genes were detected. All three isolates had a *bla*TEM-4 class A extended-spectrum beta-lactamase, *bla*OXA-1 and *bla*ACT-25 beta-lactamases. Two of the isolates (ECI02 and ECI05) carried the IncHI2 plasmid replicon, along with *bla*IMP-4, and a number of plasmid-associated antimicrobial resistance genes [6], which were missing from the carbapenem-susceptible isolate ECI03 (Table 2). This isolate (ECI03), like the two others, carried an IncL/M and colRNAI plasmid replicon, but not IncHI2, suggesting that *bla*IMP-4 was carried on the IncHI2 plasmid, whilst the other antimicrobial resistance genes were carried on IncL/M and possibly colRNAI (see Table 3).

**Table 2.** Antimicrobial resistance genes detected in all three isolates with the CARD database

	Antimicrobial resistance genes
**Genes present in all three isolates (all IncL/M- and colRNAI-positive**)	Class A broad-spectrum beta-lactamase TEM-1 Extended-spectrum beta-lactamase TEM-4 Cephalosporin-hydrolyzing class C beta-lactamase ACT-41 Oxacillin-hydrolyzing class D beta-lactamase OXA-1 Fosfomycin resistance glutathione transferase FosA Aminoglycoside N-acetyltransferase AAC(3)-IId Chloramphenicol O-acetyltransferase CatB3 Mph(A) family macrolide 2′-phosphotransferase Sulfonamide-resistant dihydropteroate synthase Sul1 Quinolone resistance pentapeptide repeat protein QnrB2 Multidrug efflux RND transporter periplasmic adaptor subunit OqxA9
**Genes not present in ECI03, but present in ECI02 and ECI05 (additionally IncH-positive**).	Metallo-beta-lactamase IMP-4 NAD(+)-rifampin ADP-ribosyltransferase Arr-3 Quinolone resistance pentapeptide repeat protein QnrA1 Aminoglycoside O-phosphotransferase APH(6)-Id Aminoglycoside O-phosphotransferase APH(3′′)-Ib Chloramphenicol O-acetyltransferase CatII Trimethoprim-resistant dihydrofolate reductase *DfrA19* Tetracycline efflux MFS transporter Tet(D) Quaternary ammonium compound efflux SMR transporter QacG2

**Table 3.** Plasmid replicons detected in the three sequenced isolates and their *bla*IMP4 status

	Antibiotic susceptibility profile	IncL/M	colRNAI	IncHI2	*bla*IMP-4 gene
**ECI02** 7 February 2018	*bla*IMP-4, CIP intermediate, MDR*	Positive	Positive	Positive	Positive
**ECI03** 18 February 2018	MDR, except cefepime-susceptible	Positive	Positive	Negative	Negative
**ECI05** 10 March 2018	*bla*IMP-4, MDR	Positive	Positive	Positive	Positive

*MDR, resistant to ciprofloxacin, gentamicin, cefepime, trimethoprim–sulphamethoxazole, tigecycline, nitrofurantoin.

## Discussion

This case contributes to the literature on the use of ceftazidime–avibactam and aztreonam combination therapy in the treatment of serious infections due to metallo-β-lactamase (MBL)-producing organisms, in the presence of other beta-lactamases. The distinguishing feature of this case is that our patient had limited surgical options for source control. Despite multiple recurrences of infection due to a persistent biliary focus over more than 7 months, and sustained treatment failure using alternative active antibiotics, the patient was successfully treated with a single limited 14-day course of CAZ–AVI–AZT treatment.

Ceftazidime–avibactam is a successful option for treatment of carbapenemase-producing enterobacterales (CPE) infections, especially those caused by *bla*KPC- and *bla*OXA-48-producing organisms. Avibactam is a beta-lactamase inhibitor with activity against Ambler class A ESBLs and carbapenemases, Ambler class C-producing AmpC beta-lactamases and *bla*OXA-48-like carbapenemases, but not MBLs [7]. Thus, the management of sepsis caused by MBL-producing CPE, such as *bla*NDM and *bla*IMP-4, remains unsatisfactory. Aztreonam is a beta-lactam antibiotic that also inhibits MBLs, and its addition to another beta-lactam antibiotic may overcome this problem [7].

Although MBLs do not hydrolyze aztreonam, which then retains activity, MBL-producing isolates may also co-produce ESBLs that confer resistance to aztreonam. The use of aztreonam–avibactam may potentially counter this, but as this drug combination is not commercially available, the combination of ceftazidime–avibactam and aztreonam has increasingly been utilized in the treatment of infection caused by MBL-producing organisms [7–10]. In this case, phenotypic detection of ESBL was confirmed by genomic analysis.

*In vitro* data using the layered Etest and chequerboard methodology has demonstrated reduction in ceftazidime–avibactam MICs by the addition of aztreonam in enterobacterales isolates with MBLs and ESBLs [11]. Isolates included in the published literature have harboured *bla*NDM or *bla*VIM alone, or in combination with *bla*OXA-48, *bla*OXA-181 or *bla*KPC-2, as well as various ESBLs. Synergy has also been demonstrated in disc diffusion assays, in agar dilution, in time–kill studies and in mouse neutropenic thigh infection models [7].

*In vivo*, the utility of this combination has been demonstrated in a prospective observational study, individual case studies and series with a range of treatment duration from 10 days to greater than 6 weeks [8]. The combination was curative in cases of *bla*NDM-1-producing *Enterobacter cloacae* and ESBL-producing *Klebsiella pneumoniae* arthroplasty infection [7], *bla*OXA-48 and *bla*NDM-1-producing persistent *Klebsiella pneumoniae* bacteraemia [9], *bla*NDM-1-producing *Pseudomonas aeruginosa* lung abscess [9] and extensive osteomyelitis due to *bla*NDM-1- and *bla*OXA-181-producing *Klebsiella pneumoniae *, which also required aggressive surgical management [12]. There was a 60 % reduction in the risk of mortality compared to treatment with other active antibiotics [10].

*bla*IMP-4-producing enterobacterales, in particular *E. cloacae* complex isolates*,* have become increasingly recognized as endemic CPE in hospitals in Australia [13–17]. One widespread hospital outbreak in the state of Queensland comprised mostly the same species as in this case, carried on an IncHI2 plasmid [16]. *bla*IMP-4-producing CPE are commonly found as environmental organisms, or may be detected as colonizing flora in hospitalized patients, particularly in the ICU or burns unit setting [13, 17]. However, once significant infections occur, potentially toxic antibiotics such aminoglycosides, colistin and tigecycline are frequently used, sometimes in combination. Once these options are exhausted, treatment choices for eradication of infection become limited.

## Conclusion

Infections due to MBLs are becoming a significant problem, with organisms producing *bla*NDM and *bla*IMP-4 causing increasing numbers of community- and healthcare-associated infections, and antibiotic treatment options are limited. Aztreonam combined with avibactam presents an increasingly useful therapeutic choice, and though currently not commercially available as a combination, the use of CAZ–AVI with aztreonam provided a safe and effective cure in this difficult biliary infection.
